# Development of a Sensitive and Fast Determination Method for Trace Carbaryl Residues in Food Samples Based on Magnetic COF (TpPa-NH_2_)@Fe_3_O_4_ Nanoparticles and Fluorescence Detection

**DOI:** 10.3390/foods11193130

**Published:** 2022-10-08

**Authors:** Juanli Du, Hao Wu, Xu Jing, Yonghe Yu, Zhisheng Yan, Jianhai Zhang

**Affiliations:** 1 College of Veterinary Medicine, Shanxi Agricultural University, Taigu 030801, China; 2 School of Chemistry and Material Science, Shanxi Normal University, Taiyuan 030001, China; 3 College of Food Science and Engineering, Shanxi Agricultural University, Taigu 030801, China

**Keywords:** magnetic solid-phase extraction, COF (TpPa-NH_2_)@Fe_3_O_4_ nanocomposite, fluorescence spectroscopy, carbaryl, food samples

## Abstract

Developing a simple and effective method for measuring carbaryl residues in food is urgent due to its widespread use and the associated health risks in agriculture, as well as various defects in existing detection techniques. The COF (TpPa-NH_2_)@Fe_3_O_4_ nanocomposite (amino modification) was synthesized via a two-step method and used as an adsorbent for the extraction of carbaryl from food samples in this study. The results indicated that COF (TpPa-NH_2_)@Fe_3_O_4_ can rapidly and successfully capture carbaryl directly from samples via π–π stacking and hydrophobic interactions, achieving maximum adsorption within 5 min under a small adsorbent quantity using a fluorescence spectrophotometer. Under the optimized conditions, carbaryl exhibited good linearity in the range of 0.2–120 µg·kg^−^^1^, and the limit of detection was 0.012 µg·kg^−1^. The recoveries of the samples were 96.0–107.4%. This method has broad application prospects for the monitoring of carbaryl in food.

## 1. Introduction

Carbaryl (1-naphthyl methylcarbamate, Sevin™) is an efficient and rapid pesticide that is popular in agricultural production [1]. However, due to improper use, carbaryl may form a residue in food products and then enter the human body through the food chain, resulting in toxicity. In human bodies, carbaryl can inhibit acetylcholinesterase (AchE), an important enzyme in the central nervous system, thereby causing serious damage to the human nervous system, muscles, liver, pancreas, and brain. Various foods can be contaminated by pesticides since they can accumulate in matrices [2]. For example, the residues of carbaryl in vegetables and fruits and the residues in bee products such as honey, which is transferred from bees in contact with carbaryl distributed in the ecological environment, will directly affect human health. Therefore, it is very important to establish analytical methods to monitor the content of carbaryl in food.

Currently, the detection methods for carbaryl and other pesticides mainly include visible spectrophotometry [3], capillary electrophoresis [4], enzyme-linked immunosorbent assay [5], gas chromatography [6], fluorescence spectroscopy [7], and high-performance liquid chromatography [8]. Among various detection techniques, fluorescence spectroscopy is one of the most convenient methods, as it has a low detection limit, high sensitivity, and fast response speed and is easy to operate [9,10]. However, carbaryl is difficult to detect directly due to its hydrophobicity, trace amounts in the environment, and matrix interference in real samples. Therefore, modern sample pretreatment methods, such as liquid–liquid extraction [11,12], solid-phase extraction [13], dispersive liquid–liquid microextraction [9,10], stir bar sorptive extraction [14], dispersive solid phase extraction [3], and cloud point extraction [4], are commonly used in sample preparation to detect carbaryl from complex matrices. However, these methods still have some shortcomings, such as limited diffusion and high labor intensity, the use of a large amount of solvents that are toxic to the human body, and in some cases, the inability to process a large number of samples [15]. This leads to continuous efforts to develop new methods to overcome their shortcomings.

Magnetic solid phase extraction (MSPE) is similar to dispersive solid phase extraction, except that an external magnet is required for extraction. It is widely used in sample pretreatment due to its high extraction efficiency and ease of operation [16]. Adsorbents play an important role in MSPE by simplifying the sample pretreatment process through magnetic separation and directly affecting the extraction performance [17]. To date, a variety of magnetic adsorbents have been developed for the extraction and enrichment of carbaryl, including Fe@Ag [18], magnetic o-hydroxyazobenzene [19], graphene–carbon nanosphere composites [20], and magnetic molecular imprinted polymers [21]. Nevertheless, new adsorbents with good adsorption performance and chemical stability should be developed to meet the requirements of analytes.

Covalent organic frameworks (COFs) are ordered crystalline porous polymers composed of light elements (*C*, *H*, *O*, *N*, *B*, etc.) and organic monomers through strong covalent bonds [22]. However, COFs are highly hydrophobic and difficult to separate from water due to their low density, limiting their applications [23]. Magnetic covalent organic frameworks (MCOFs) combined with the advantages of COFs and magnetic nanoparticles have attracted widespread attention and can be used as ideal adsorbents for MSPE [24]. MCOFs can offer large specific surface area, strong magnetic properties and chemical stability; furthermore, they can rapidly separate contaminants from solutions, thereby compensating for the disadvantages of COFs. Thus, MCOFs have shown promising applications as adsorbents in many fields, especially in sample pretreatment. Fe_3_O_4_ is the most popular composite material owing to its easy synthesis, low cost, and excellent magnetic properties, which make it highly workable [25]. MCOF is formed by combining COF with Fe_3_O_4_; the resulting spherical Fe_3_O_4_ offers a homogeneous growth environment for COF growth, and COF@Fe_3_O_4_ is rich in functional groups and easy to apply in separation methods [26]. For example, Liu et al. explored 1,3,5-triformylphloroglucinol (Tp) and benzidine as structural units to synthesize COF@Fe_3_O_4_ and used them to detect sulfonamides in food samples [27]. Wu et al. used 1,3,5-triformylphloroglucinol and ethidium bromide on an Fe_3_O_4_ surface and prepared EB-COF@Fe_3_O_4_ for the determination of benzoylurea insecticides in water, juice, and tomato [28]. More targeted MCOFs with specific functional sites have been investigated extensively, and some research results have been obtained in recent years, but different detection requirements of different targets have not been resolved to date. The preparation of different MCOFs for different target analytes is very important in the field of detection and analysis.

Therefore, this paper reports the fabrication and utilization of COF (TpPa-NH_2_)@Fe_3_O_4_ nanocomposites as adsorbents and, for the first time, combines them with a fluorescence spectrophotometer for the analysis of carbaryl in food samples, which greatly simplifies the sample preparation procedure and improves the analytical throughput. We synthesized a chemically stable β-ketoenamine COF (TpPa-NO_2_), anchoring nitro groups in the pores, and then an amine functionalized COF (TpPa-NH_2_) was obtained through the reduction of the nitro groups. After reducing the nitro group to an amino group, the amino group is an electron-donating group that can enhance the activation of the benzene ring to COF (TpPa-NH_2_)@Fe_3_O_4_, and it is easier to bind carbaryl through π–π interaction. COF (TpPa-NH_2_)@Fe_3_O_4_ shows high chemical stability and magnetic properties in acidic and alkaline environments. The introduction of amines into COFs makes the method more attractive and novel, as amines are one of the active groups that are currently widely used for the further modification of materials. Amino-modified COF materials are widely used and are promising in selective adsorption processes. Compared with the traditional method, this method has the advantages of being easy to operate, high speed, low cost, and environmentally friendly, with a high enhancement factor, a high sensitivity, and good selectivity and stability. In order to better elucidate the adsorption mechanism of carbaryl using COF (TpPa-NH_2_)@Fe_3_O_4_, the extraction performance of COF(TpPa-NH_2_)@Fe_3_O_4_, for different types of pesticides (including carbaryl, metoxuron, isoproturon, carbofuran, and propoxar), was studied. Magnetic solid phase extraction was carried out using the same method, and a comparative analysis was performed through HPLC (Table 1). The results showed that COF(TpPa-NH_2_)@Fe_3_O_4_ had the highest extraction efficiency for carbaryl, while the extraction efficiency of other pesticides was only approximately 60%. The π–π stacking and hydrophobic interaction between COF(TpPa-NH_2_)@Fe_3_O_4_ and carbaryl are the main reasons for adsorption. The objective of this study is to develop a reliable and fast analytical approach for food safety analysis in the agricultural and food chemical fields.

## 2. Materials and Methods

### 2.1. Apparatus

Fluorescence signals were measured using a Cary Eclipse fluorescence spectrophotometer (Agilent Technologies, Palo Alto, CA, USA). The HPLC equipment (Waters, Milford, MA, USA) consisted of an online filter and a 1525 binary high-performance liquid pump (used to transfer the mobile phase for analysis). Deionized water in the experiment was purified using a Milli-Q water system (Millipore, Bedford, MA, USA). The buffered solution was adjusted with a pH meter (Model pHS-3C, Shanghai J & K Science, Shanghai, China). A KQ-500DV-type ultrasonic scrubber (Kunshan Hechuang, Suzhou, China) was used to assist adsorption and desorption.

### 2.2. Chemicals and Reagents

Iron (III) chloride hexahydrate (FeCl_3_·6H_2_O) and sodium acetate (CH_3_COONa) were obtained from Thermo Fisher Scientific (Shanghai, China). Ethylene glycol ((CH_2_OH)_2_) and ethylenediamine ((CH_2_NH_2_)_2_) were purchased from Kermel Chemical Reagent (Tianjin, China). Britton–Robinson buffer (B-R, 0.04 mol/L) solution, 1,3,5-Triformylphloroglucinol (Tp), p-phenylenediamine (Pa-1), 2-nitro-P-phenylenediamine (Pa-NO_2_), and 4-chloro-m-phenylenediamine (Pa-Cl) were purchased from Aladdin (Shanghai, China). Dimethyl sulfoxide (DMSO), tetrahydrofuran (C_4_H_8_O), methanol (CH_3_OH), and other solutions were analytical grade reagents. Magnesium sulfate, sodium chloride, and sodium citrate were all analytical grade reagents.

Carbaryl was produced by Aladdin Biochemical Technology Co., Ltd. (Shanghai, China). Carbaryl stock solution (0.1 mg·mL^−^^1^) was prepared with ethanol as solvent; the stock solution was diluted with ethanol to obtain the working solution [29]. Pesticide standards (metoxuron, isoproturon, carbofuran, and propoxar) were purchased from Aladdin reagent (Shanghai, China). All honey and apple samples were purchased from a local supermarket in Shanxi Province. Cabbage was obtained from the experimental farms of Shanxi Agricultural University (Taigu, China).

### 2.3. Preparation of Fe_3_O_4_ Magnetic Nanoparticles

The preparation of Fe_3_O_4_ was performed according to the literature [30]. FeCl_3_·6H_2_O (1 g) and 20 mL (CH_2_OH)_2_ were placed in beakers and sonicated. Then, CH_3_COONa (3 g) was slowly added to the beaker. This was stirred for 30 min to form a yellow solution. After that, 10 mL (CH_2_NH_2_)_2_ was added, which formed a transparent homogeneous solution after 30 min. Then, the mixture was sealed in a 50 mL Teflon-lined stainless steel autoclave. The autoclave was heated and maintained at 200 °C for 8 h and then cooled to room temperature. The resulting black product was washed several times with water and ethanol, separated by an external magnetic field, and dried in a vacuum oven at 60 °C.

### 2.4. Preparation of COF @Fe_3_O_4_ Magnetic Nanoparticles

COF (TpPa-1)@Fe_3_O_4_ and COF (TpPa-NO_2_)@Fe_3_O_4_ were prepared following previous studies [28,31]. COF (TpPa-Cl)@Fe_3_O_4_ and COF (TpPa-NH_2_)@Fe_3_O_4_ were prepared through a stirring and solvothermal method.

COF (TpPa-Cl)@Fe_3_O_4_ was prepared by stirring at room temperature. Fe_3_O_4_ (120 mg) was weighed and placed in 100 mL dimethyl sulfoxide (DMSO). Then, the DMSO solution was treated via sonication for 10 min to disperse the Fe_3_O_4_. Tp (120 mg), and Pa-Cl (96.26 mg) was added in turn, and the solution was sonicated for 10 min after each addition. Glacial acetic acid (2 mL) was added dropwise under electric stirring, and the reaction was conducted for 30 min and then left for 30 min to produce a brownish–yellow product. The product was washed several times with methanol and water until the supernatant was almost colorless. The magnetic composite was dried in a vacuum oven for 12 h.

COF (TpPa-NH_2_)@Fe_3_O_4_ was prepared according to a method reported in the literature [32]. First, 100 mg of prepared COF (TpPa-NO_2_)@Fe_3_O_4_ was weighed, dispersed in a round bottom flask containing 5 mL of anhydrous tetrahydrofuran, and sonicated for 10 min; then, 1 g of SnCl_2_·2H_2_O was added, and the resulting product was ultrasonicated for 10 min before being placed in a water bath, heated to 75 °C and refluxed for 3 h. The precipitate was separated and washed with HCl (0.1 mol·L^−1^) water and acetone. The product was dispersed in 30 mL of a mixture containing 1,3,5-trimethylbenzene and 1,4-dioxane, kept at 120 °C for 24 h, and cooled naturally to produce the magnetic nanomaterial COF (TpPa-NH_2_)@Fe_3_O_4_. Subsequently, the product was washed with 100 mL acetone and placed in a vacuum drying oven to dry.

### 2.5. Real Sample Preparation

A certain amount of the cabbage and apple samples was cut into small pieces and homogenized with a blender. Homogenized samples (5 g) were accurately weighed and placed in centrifuge tubes. Twenty milliliters of methanol was added and mixed with ultrasonication for 10 min. After centrifugation at 4000 rpm for 5 min, the supernatants were collected and filtered through a 0.22 μm filter to detect carbaryl in the sample matrix using the proposed strategy (in triplicate).

A 5 g sample of honey was dissolved in 20 mL ultrapure water to reduce its viscosity. The diluted sample solution was then filtered with a 0.22 μm filter membrane for the MSPE process (in triplicate). The homogenates of the above three samples were stored in a refrigerator at 4 °C.

### 2.6. MSPE Procedure

The MSPE procedure was carried out as follows: COF @Fe_3_O_4_ (4 mg) was added to 50 mL working solution containing carbaryl. The pH was adjusted to 6 by adding Britton–Robinson (B–R) buffer. The solution was sonicated for 5 min to uniformly disperse the COF@Fe_3_O_4_, and the supernatant was decanted under the action of an external magnetic field. The collected magnetic adsorbent was dried with a gentle stream of nitrogen gas. It was then desorbed by sonication with 0.4 mL acetonitrile and B–R buffer (1:1, *v*/*v*, pH = 4) for 6 min, and the magnet was placed next to the colorimetric tube again. The supernatant was collected under a magnetic field and transferred to a quartz cuvette. Finally, the relative fluorescence intensity (RFI) of the carbaryl was measured.

### 2.7. Optimization of MSPE Process

An experimental study was conducted to determine the maximum carbaryl extraction efficiency of the magnetic materials. The key extraction parameters included the choice of magnetic material COF @Fe_3_O_4_, and the magnetic materials COF (TpPa-1)@Fe_3_O_4_, COF (TpPa-Cl)@Fe_3_O_4_, COF (TpPa-NH_2_)@Fe_3_O_4_, and COF (TpPa-NO_2_)@Fe_3_O_4_ were compared. The pH of the extraction solution and the adjusted pH of 3–10 were compared to determine which pH condition has the best extraction effect. For the amount of magnetic adsorbent, 2–9 mg was chosen to determine the optimal amount of adsorbent. Additionally, the extraction and desorption times were determined. The adsorption conditions included the selection and volume of the adsorption solvent (methanol, ethanol, acetonitrile, and isopropanol) and the optimal pH value (3–10) of the adsorption solvent. All the experiments were performed in triplicate, and the averaged results were applied in data analysis. One variable was changed at a time, other conditions were unchanged, and the optimal value was selected as the basis for subsequent experiments. The fluorescence intensity reflects the extraction effect. The higher the fluorescence intensity, the better the extraction effect is.

## 3. Results and Discussion

### 3.1. Optimization of the Extraction and Desorption Conditions

The best extraction efficiency of carbaryl by magnetic materials was determined in the experiment. Extraction conditions were the type and amount of adsorbent, solution pH, and extraction time, and the desorption conditions were the solution type, volume, pH, time, and ratio of solvent to BR buffer. One variable was changed at a time, and the best value was selected as the basis for subsequent experiments.

#### 3.1.1. Selection of Magnetic Material

**Figure 1:**
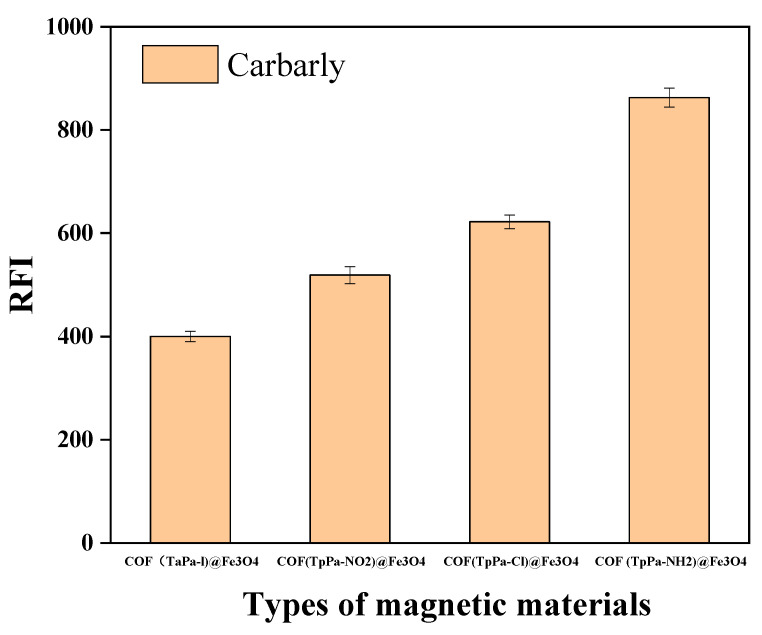
Selection of magnetic material. To conclude, COF (TpPa-NH_2_) @Fe_3_O_4_ was the best extractant (50 mL of sample solution, c = 100 ng·mL^−1^, extraction time: 5 min, desorption time: 6 min, *n* = 3).

Four magnetic materials, COF (TpPa-1)@Fe_3_O_4_, COF (TpPa-Cl)@Fe_3_O_4_, COF (TpPa-NH_2_)@Fe_3_O_4_, and COF (TpPa-NO_2_)@Fe_3_O_4_, were compared with respect to carbaryl extraction under identical conditions. As shown in Figure 1, COF (TpPa-NH_2_)@Fe_3_O_4_ had the optimal extraction effect. After the reduction of nitro to amine, the affinity of the framework for carbaryl was greatly increased. The amino group is an electron-donating group that can enhance the activation effect of the benzene ring on COF (TpPa-NH_2_)@Fe_3_O_4_, and it is easier to bind carbaryl via π–π interactions. The chlorine and nitro groups contained in COF (TpPa-Cl)@Fe_3_O_4_ and COF (TpPa-NO_2_)@Fe_3_O_4_ could passivate the benzene ring, making it less reactive and weaker in the π–π binding, while also weakening the extraction of carbaryl. In conclusion, the highest extraction recovery was obtained using the COF (TpPa-NH_2_)@Fe_3_O_4_, and it was used as the adsorbent in all subsequent experiments.

#### 3.1.2. Effect of Sample Solution pH

**Figure 2:**
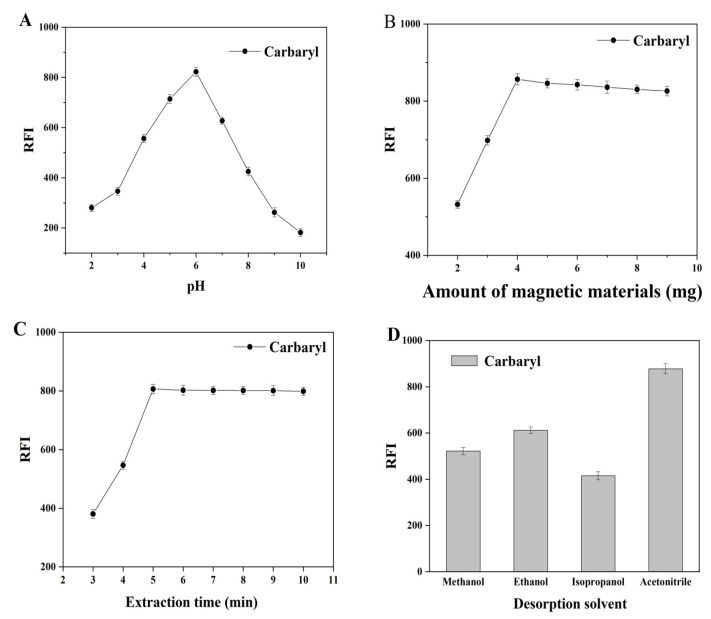
Optimization of the MSPE method (50 mL of sample solution, c = 100 ng·mL^−1^, *n* = 3): (**A**) effect of sample solution pH; (**B**) effect of adsorbent amount; (**C**) effect of the extraction time; and (**D**) effect of desorption solvent type.

In MSPE, the pH of the sample solution is an important factor because it directly determines the form in which the analytes are present and further affects their extraction efficiency [19]. Therefore, the pH value of the sample solution was adjusted to a suitable value to ensure that the analytes existed in the appropriate form and achieved effective adsorption. At high pH (pH > 10), carbaryl becomes unstable under alkaline conditions, and the molecular structure is easily disrupted [33]. Therefore, the pH of the sample solution was investigated under pH values of 3–10, as shown in Figure 2A. The extraction rate of carbaryl increased when the pH of the sample increased from 3 to 6, although it decreased significantly when the pH increased from 6 to 10. These results are in agreement with those of a previous study [34]. Carbaryl was unstable and decomposed under alkaline conditions. When the solution was in the acidic range, carbaryl mainly existed in the form of intermediates, and there were many π–π interactions with magnetic COF (TpPa-NH_2_)@Fe_3_O_4_, so the extraction recovery was higher. The results indicate that adsorption experiments were performed at the chosen pH of 6.

#### 3.1.3. Amount of Magnetic Adsorbent

The adsorbent dosage has a significant effect on MSPE efficiency. This effect was observed by varying the dosage of COF (TpPa-NH_2_)@Fe_3_O_4_ from 2 to 9 mg, with other conditions remaining unchanged. As shown in Figure 2B, when the dosage of adsorbent increased from 2 to 4 mg, the extraction rate gradually increased. However, the extraction efficiency showed little change when the dosage exceeded 4 mg. Thus, 4 mg magnetic COF (TpPa-NH_2_)@Fe_3_O_4_ was selected for carbaryl extraction to ensure an adequate extraction efficiency.

#### 3.1.4. Effect of the Extraction and Desorption Time

As MSPE is a time-dependent equilibrium process, the extraction time represents another important influencing factor, and a suitable extraction time is essential for ensuring adequate contact between the adsorbent and analyte. The extraction time for carbaryl varied from 3 to 10 min, as shown in Figure 2C. When the time was increased from 3 to 5 min, the extraction of carbaryl gradually increased, indicating sufficient adsorption sites for COF (TpPa-NH_2_) @Fe_3_O_4_ in the initial process. When the extraction time increased, the extraction remained essentially unchanged, indicating that the adsorption equilibrium was reached within 5 min. Therefore, 5 min was chosen as the extraction time in subsequent experiments. Furthermore, the effect of desorption time on the extraction effect was explored, and the results show that the extraction effect was optimal for a desorption time of 6 min, when the analytes were almost completely eluted.

#### 3.1.5. Optimization of Desorption Conditions

The desorption steps also play an important role in the MSPE process. Three experimental parameters, the type, volume, and pH of the desorption solvent, were optimized. Four solvents (methanol, ethanol, acetonitrile, and isopropanol) were examined, and the results are shown in Figure 2D. Acetonitrile had the best desorption effect, so acetonitrile was chosen as the desorption solvent. A large volume of liquid leads to a decrease in detection sensitivity via the dilution effect; hence, the volume of the desorption solvent was further optimized. To obtain a high sensitivity, 0.2 mL acetonitrile was used for desorption. The pH of the desorption solvent was adjusted using the B–R buffer. According to the literature, carbaryl with a pKa value of approximately 12 shows weak alkalinity and will be hydrolyzed under alkaline conditions [33]. The effect of pH on the desorption of carbaryl was investigated by adjusting the pH from 3 to 10. The results showed that the extraction of samples with pH values under acidic conditions of pH = 4 was conducive to the desorption of carbaryl. The optimal extraction recovery was achieved at a 1:1 ratio of acetonitrile to B–R buffer (pH = 4).

### 3.2. Reusability of the Magnetic Adsorbent

**Figure 3:**
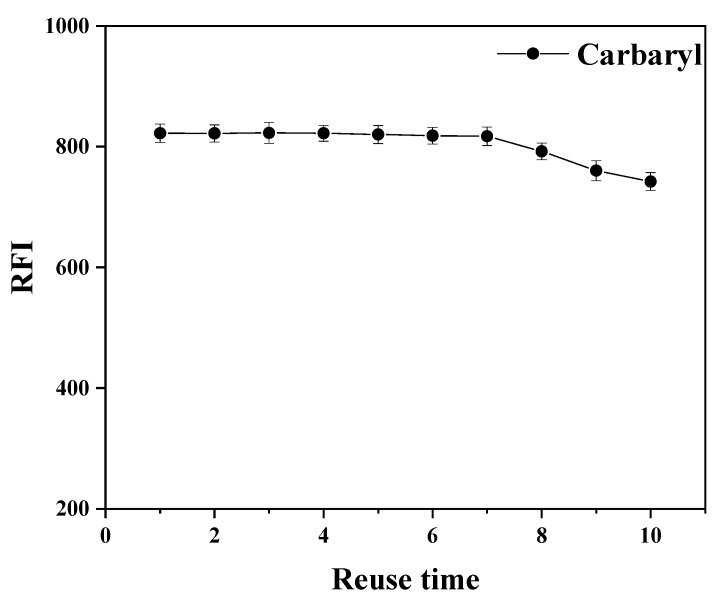
Reusability of the magnetic COF (TpPa-NH_2_)@Fe_3_O_4_ (5 mg COF (TpPa-NH_2_)@Fe_3_O_4_, 50 mL of sample solution; c = 100 ng·mL^−1^; extraction time: 5 min; 0.4 mL acetonitrile as desorption solvent was vortexed for 6 min, *n* = 3).

Reusability is an important factor for evaluating the efficiency of a magnetic adsorbent. As the durability and lifetime of the adsorbent can be evaluated from its reusability and stability, we investigated the number of repetitions of COF (TpPa-NH_2_)@Fe_3_O_4_. After repeating the sorption–desorption cycle experiment 10 times, the results (Figure 3) showed that the adsorbent could be repeatedly used at least 7 times without affecting its carbaryl extraction effect. This indicates that COF (TpPa-NH_2_)@Fe_3_O_4_ exhibits good reusability and stability under repeated usage.

### 3.3. Excitation and Emission Spectra

**Figure 4:**
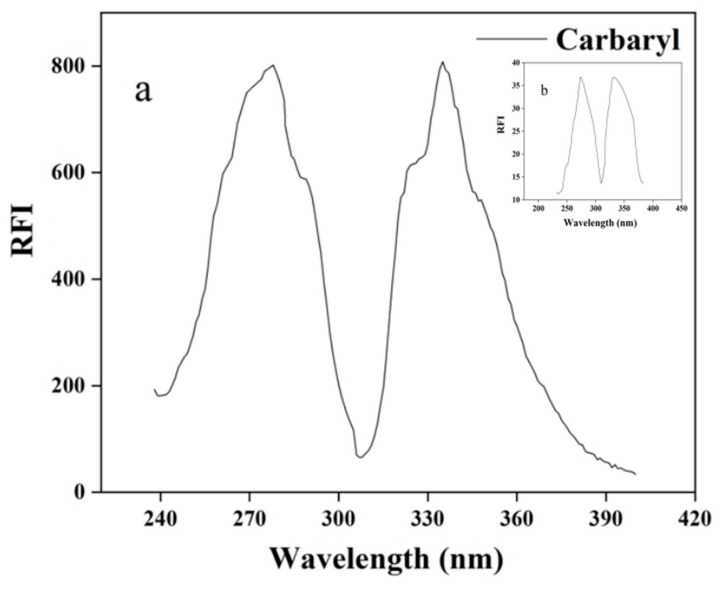
Excitation and emission spectra: (**a**) excitation and emission spectra of carbaryl desorbed; (**b**) excitation and emission spectra of carbaryl before extraction.

The excitation and emission spectra of carbaryl were measured using a fluorescence spectrophotometer (Figure 4). The resulting spectra were found at 276 and 335 nm, respectively; thus, these two wavelengths were selected as excitation and emission conditions because the reagent blank had no influence on the drug determination process.

### 3.4. Adsorption Mechanism

In order to study the possible adsorption mechanism of COF (TpPa-NH_2_)@Fe_3_O_4_ for analytes, different pesticide compounds (carbaryl, metoxuron, isoproturon, carbofuran, and propoxar) were comparatively evaluated and determined using HPLC equipment (Table 1). All experiments were carried out under the most suitable conditions. The octanol/water partition coefficient (logKow) is an important index of hydrophobicity. The hydrogen bond acceptor and donor indicate a hydrogen bond shift, and the high extraction recoveries of the analytes indicate a high affinity between COF (TpPa-NH_2_)@Fe_3_O_4_ and analytes. COF (TpPa-NH_2_)@Fe_3_O_4_ had a large specific surface area and, therefore, provided a large number of adsorption sites. Moreover, COF (TpPa-NH_2_)@Fe_3_O_4_ featured numerous benzene rings. As shown in Table 1, the highest extraction recovery of carbaryl was 99.5%. In terms of the molecular structure, the conjugation system of carbaryl exceeded that of the other compounds, indicating that the π–π stacking between COF (TpPa-NH_2_)@Fe_3_O_4_ and carbaryl plays an important role in extraction. By comparing several pesticides, carbaryl was found to have a higher logKow value and extraction recovery, suggesting that hydrophobic interactions also had an effect on sorption. Although isoproturon had a higher logKow value, its extraction recovery (76.1%) was lower than that of carbaryl, further confirming that π–π stacking interactions between the analyte and COF (TpPa-NH_2_)@Fe_3_O_4_ play a major role in extraction. The number of hydrogen bond donor–acceptors reflects the preference for hydrogen bonding, and the extraction effect is not significant. The above experimental results show that π–π stacking and hydrophobic interactions are the two main factors affecting extraction, and π–π stacking plays a dominant role [34,35].

### 3.5. Analytical Performance

The linearity, limit of detection (LOD), limit of quantification (LOQ), and precision were determined under the optimal extraction conditions described above. Several factors were evaluated to determine the suitability of the method for carbaryl extraction. The results are summarized in Table 2. The LOD and LOQ were calculated according to the equations 3 σ/K and 10 σ/K, respectively, where K is the slope of the calibration curve and σ is the relative standard deviation of the blank samples (11 measurements). The values of LOD and LOQ were 0.012 and 0.2 µg·kg^−^^1^, respectively. The calculated LOD value is much lower than the maximum residue limits of carbaryl according to the European Union (0.05 ug·mL^−1^) and the United States (0.02 ug·mL^−1^) pesticide database [36], as well as the detection methods of carbaryl reported in many other studies. Satisfactory calibration curves were obtained within the range of 0.2–120 µg·kg^−^^1^, and a satisfactory linear correlation coefficient (R^2^) of 0.9997 was obtained, indicating good linearity. The precision and recovery of the method were measured. The precision of the method was assessed by calculating the relative standard deviation (RSD) of the assay signals at three concentrations of 2, 50, and 100 µg·kg^−^^1^. They were calculated on the same day (*n* = 6) and on different days (*n* = 6). The results showed that the RSDs were ≤3.3% for the intraday and interday precisions. A stability study was performed by analyzing the samples spiked at a concentration of 100 µg·kg^−1^ (for each analyte) and storing them for 24 h at various temperatures, including 8, 23 (room temperature), and −19 °C [37]. A series of comparisons between the results obtained and the newly prepared samples showed no change, indicating that the samples were stable.

#### Analysis of Food Samples

In order to verify the feasibility of this method, magnetic COF (TpPa-NH_2_)@Fe_3_O_4_ was used as the extractant, and carbaryl was not detected in the original food samples. So, a standard addition method was used to determine the content of carbaryl in the food samples. According to the Codex Alimentarius: “Guidelines on performance criteria for methods of analysis for the determination of pesticide residues in food and feed”, acceptable mean recoveries for enforcement purposes should normally range from 70 to 120% with RSD ≤ 20%. The food samples were complex and required additional pretreatment prior to the MSPE process. No significant interference peaks were observed. The results are shown in Table 3. The recovery rate of the food samples was 96.0–107.4%. The results indicate that the method can be used to successfully detect carbaryl in real samples, and there is no significant difference between the concentration measured in our experiment and the concentration of the added standard.

In order to verify the applicability and accuracy of the method, the content of carbaryl in honey cabbage and apple samples was determined according to the Chinese National Standard “Determination of Pesticides in Food” (GB 23200.121-2021). The results were compared with the proposed method. The QuEChERS method and LC–MS/MS detection were used in the Chinese National Standard for Determination of Pesticides in Food. A certain amount of the samples was placed into a 50 mL centrifuge tube, and then acetonitrile was added, followed by magnesium sulfate, sodium chloride, and sodium citrate, before centrifugation at 4200 rpm for 5 min after intense shaking. The supernatant was placed in a centrifuge tube containing magnesium sulfate adsorbent and primary secondary amine (PSA); then, the tube was shaken violently for half a minute and centrifuged at 4000 rpm for 5 min, and the solution was filtered through a 0.45 μm filter. LC–MS/MS was used for detection. The results showed that there was no significant difference between the two methods, and no carbaryl was detected in the blank food samples. Statistical analysis was performed using SPSS 16.0 (Chicago, USA). A paired Student’s *t*-test was used to evaluate significant differences; the results showed no significant difference.

### 3.6. Comparison with Other Reported Methods

The method was compared with previous methods for the extraction and determination of carbaryl, and the results are shown in Table 4. Magnetic COF (TpPa-NH_2_)@Fe_3_O_4_ combined with fluorescence analysis was reported for the first time for the determination of carbaryl in food, and a good extraction effect was obtained. The magnetic material COF (TpPa-NH_2_)@Fe_3_O_4_ MSPE method is better than the other methods in terms of LOD, relative standard deviations, linearity, and extraction time. Compared with other pretreatment methods (e.g., solid-phase and liquid–liquid extraction), the extraction and desorption processes of this method could be completed in a shorter time using an external magnetic field. Magnetic COF materials have the advantages of simple preparation, low cost, and environmental friendliness. Only 4 mg of adsorbent was used to extract carbaryl, and the extraction efficiency was good.

## 4. Conclusions

An effective method using COF (TpPa-NH_2_)@Fe_3_O_4_ as the adsorbent for the detection of carbaryl in food samples based on MSPE technology and fluorescence spectrophotometry was first established. Unlike previously used sorbents, this synthesis of COF (TpPa-NH_2_)@Fe_3_O_4_ adsorbents does not require any expensive raw materials, which is crucial in separation sciences. The method has the advantages of a wide linear range, low LODs, good accuracy and reproducibility, and high recovery. Compared with other extraction methods, this method can extract carbaryl efficiently in a very short time with only 4 mg of adsorbent. The prepared COF (TpPa-NH_2_)@Fe_3_O_4_ not only has good preparation reproducibility and chemical stability but can also be reused many times. The method can not only provide a powerful reference for methodological evaluation, but can also be used for the adsorption and removal of trace pollutants in other complex matrices in future studies.

## Figures and Tables

**Table 1:** Adsorption of other pesticide with magnetic COF (TpPa-NH_2_)@Fe_3_O_4_.

Analytes	Structure	Molecular Weight	H Bond Acceptors	H Bond Donors	LogK_ow_ ^a^	ER ^b^ (%)
Carbaryl	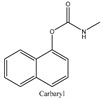	200.21	2	1	1.85	99.5
Metoxuron	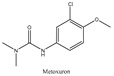	228.68	4	1	1.64	55.2
Isoproturon	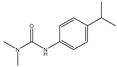	206.28	3	1	2.87	76.1
Carbofuran	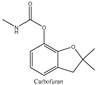	221.25	4	1	1.52	47.6
Propoxur	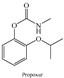	209.2	4	1	1.90	67.3

All the experiments were performed with 50 mL water solution spiked with 100 ng·mL^−1^ of each of the pesticides; the amount of the COF (TpPa-NH_2_)@Fe_3_O_4_ was 4 mg; 0.4 mL desorption solvent was used to desorb the analytes. ^a^ K_ow,_ octanol/water partition coefficient; ^b^ ER, extraction recovery.

**Table 2:** Analytical performance and parameters of the method.

Analytes	Linear Equation (µg·kg^−1^)	Linear Range (µg·kg^−1^)	R^2^	LOD (µg·kg^−1^)	RSD (%)					
					Intra-Day Precision at the Concentrations of (µg·kg^−1^)			Inter-Day Precision at the Concentrations of (µg·kg^−1^)		
					2	50	100	2	50	100
Carbaryl	y = 7.42c + 36.2	0.2–120	0.9997	0.012	1.6	2.2	2.5	2.0	2.6	3.3

**Table 3:** Determination of carbaryl in food samples (*n* = 5).

Sample	Original (µg·kg^−1^)	Added (µg·kg^−1^)	Found (µg·kg^−1^)	Recovery (%)	RSD (%) (*n* = 3)
Honey	ND	1	1.02	102.3	2.5
		15	15.76	105.1	1.9
		30	29.86	99.5	3.2
Cabbage	ND	1	1.07	107.4	1.6
		15	15.72	104.8	3.1
		30	30.22	100.7	1.8
Apple	ND	1	0.96	96.0	3.6
		15	15.12	100.8	2.2
		30	29.89	99.6	2.6

The tabulated value of *t* at the 95% confidence limit is *t* = 2.32. ND: not detected; RSD: relative standard deviation.

**Table 4:** Comparison to previously proposed methods.

Extraction Method	Technique	Analytes	Sample	Extraction Time (min)	Linear Range (µg·kg^−1^)	LOD (µg·kg^−1^)	RSD (%)	Reference
CPE	Visible Spectrophotometry	Carbaryl	Six vegetables	4	100–7000	50	2.3	[3]
DLLME	HPLC	Carbaryl	Cucumber Spinach	250	1–500	0.3–1	-	[8]
LLE	LC-MS	Carbaryl	honeybees	13	4–9	3	≤14	[11]
LLE	HPLC-UV	Carbaryl	Drinking Water	10	0.005–0.01	0.001	4.6	[12]
SPE	HPLC	Carbaryl	Water	-	0.01	0.01	1.8	[13]
SBSE	HPLC	Carbaryl	Water Samples	23	0.002–30	0.0003	3.3–4.5	[14]
MSPE	Fluorimetry	Carbaryl	Honey cabbage apple	5 ^a^	0.2–120	0.012	1.6–3.6	This work

^a^: Synthesis time of COF (TpPa-NH_2_)@Fe_3_O_4_, a = 27 h; CPE, cloud point extraction; DLLME, dispersive liquid–liquid microextraction; LLE, liquid–liquid extraction; SPE, solid phase extraction; SBSE, stir bar sorptive extraction; MSPE magnetic solid phase extraction; HPLC, high performance liquid chromatography; LC-MS, liquid chromatograph-mass spectrometer; HPLC-UV, high performance liquid chromatography with ultraviolet detection.

## Data Availability

Data is contained within the article.
